# The inpatient hospital burden of comorbidities in HCV-infected patients: A population-based study in two Italian regions with high HCV endemicity (The BaCH study)

**DOI:** 10.1371/journal.pone.0219396

**Published:** 2019-07-10

**Authors:** Simona Cammarota, Anna Citarella, Antonella Guida, Valeria Conti, Teresa Iannaccone, Maria Elena Flacco, Francesca Bravi, Cristina Naccarato, Antonella Piscitelli, Raffaele Piscitelli, Alfredo Valente, Giulio Calella, Nicola Coppola, Giustino Parruti

**Affiliations:** 1 LinkHealth Health Economics, Outcomes & Epidemiology s.r.l., Naples, Italy; 2 Directorate-General for Protection of Health, Campania Region, Naples, Italy; 3 Department of Medicine, Surgery and Dentistry “Scuola Medica Salernitana”, University of Salerno, Baronissi (SA), Italy; 4 Regional Healthcare Agency of Abruzzo, Pescara, Italy; 5 “Sant’Anna” University Hospital of Ferrara, Ferrara, Italy; 6 Italian National Agency for New Technologies, Energy and Sustainable Economic Development “ENEA”, Bologna, Italy; 7 Specialisation School, Department of Pharmacy, University of Salerno, Fisciano, Italy; 8 Specialisation School, Department of Pharmacy, University of Naples “Federico II”, Naples, Italy; 9 Infectious Diseases Unit, Pescara General Hospital, Pescara, Italy; 10 Infectious Diseases Unit, AORN Caserta, University of Campania “Luigi Vanvitelli”, Caserta, Italy; Centers for Disease Control and Prevention, UNITED STATES

## Abstract

**Background & aims:**

Hepatitis C (HCV) is associated with several extrahepatic manifestations, and estimates of the hospitalization burden related to these comorbidities are still limited. The aim of this study is to quantify the hospitalization risk associated with comorbidities in an Italian cohort of HCV-infected patients and to assess which of these comorbidities are associated with high hospitalization resource utilization.

**Methods:**

Individuals aged 18 years and older with HCV-infection were identified in the Abruzzo’s and Campania’s hospital discharge abstracts during 2011–2014 with 1-year follow-up. Cardio-and cerebrovascular disease, diabetes and renal disease were grouped as HCV-related comorbidities. Negative binomial models were used to compare the hospitalization risk in patients with and without each comorbidity. Logistic regression model was used to identify the characteristics of being in the top 20% of patients with the highest hospitalization costs (high-cost patients).

**Results:**

15,985 patients were included; 19.9% had a liver complication and 48.6% had one or more HCV-related comorbidities. During follow-up, 36.0% of patients underwent at least one hospitalization. Liver complications and the presence of two or more HCV-related comorbidities were the major predictors of hospitalization and highest inpatient costs. Among those, patients with cardiovascular disease had the highest risk of hospitalization (Incidence Rate Ratios = 1.42;95%CI:1.33–1.51) and the highest likelihood of becoming high-cost patients (Odd Ratio = 1.37;95%CI:1.20–1.57).

**Conclusion:**

Beyond advanced liver disease, HCV-related comorbidities (especially cardiovascular disease) are the strongest predictors of high hospitalization rates and costs. Our findings highlight the potential benefit that early identification and treatment of HCV might have on the reduction of hospitalization costs driven by extrahepatic conditions.

## Introduction

Chronic infection with the hepatitis C virus (HCV) is an important public health challenge with a significant economic burden worldwide [1–3]. In Western Europe, Italy has among the highest number of HCV-infected patients (HCV prevalence 1.1%) and the highest rate of death from HCV-related hepatic complications (19.4% − 42.70% death rate of carcinoma) [2, 4].

Overall, 30% of the HCV patients chronically infected may progress to cirrhosis in their lifetime, whereas 3%–8% of cirrhotic patients may develop hepatocellular carcinoma (HCC) [5, 6]. The progression to cirrhosis is often clinically silent, and many HCV patients did not come forward until cirrhosis, liver decompensation or HCC occurred [7–9]. The advanced stages of decompensated cirrhosis (DC) or HCC usually result in high healthcare cost, especially due to hospital admissions [10–12].

Besides the hepatic manifestations, extrahepatic conditions have been reported in more than 70% of patients living with chronic HCV infection [13–16]. Among them, the majority of available data concern HCV-related autoimmune and/or lymphoproliferative disorders [17, 18]. Several non-infectious disorders like diabetes, cardio- and cerebrovascular events as well as renal impairment due to cryoglobulinemia have been recently linked to chronic HCV infection [16, 18–20]. All these conditions lead to increased hospitalization rates, up to 13% per year [21].

Recently, the treatment of chronic HCV infection has been revolutionized by the development of direct-acting antiviral agents (DAAs). DAA regimens yield sustained virological response (SVR) rates of approximately 95%, even in difficult-to-treat patients, such as patients with advanced liver disease, cirrhosis or chronic kidney disease [22–26]. Achievement of SVR has the potential benefits of risk reduction of liver disease progression, leading to improved long-term clinical outcomes and health-related quality of life [27, 28]. Therefore, it may be relevant to reduce the impact of liver and extrahepatic manifestations within state-run policies of HCV screening and treatment. In particular, the potential cost-benefit of such policies is gaining growing attention by States and Insurance payers in Italy, as many recent reports document that the costs of chronic HCV infection increase with disease progression [29–32].

Improving the knowledge of patient characteristics that may influence the hospitalization resource use and inherent costs is therefore a mandatory task in the present worldwide scenario, and may help the decision-makers to manage HCV infection through early treatment or efficient screening practices (e.g., test and treat therapies) and prioritize patients with a potential high need for DAA treatment [2, 11, 33]. In order to assess the historical baseline against which future economic outcomes can be gauged, it is also relevant to examine the distribution of health care costs coming from the pre-DAAs era. Nevertheless, little is still known about the extent at which costs are skewed in HCV-infected patients and, importantly, whether there are observable characteristics associated with patients who are likely to utilize more health resources.

The present study aims to quantify the hospitalization risk associated with comorbidities in a large cohort of Italian patients admitted to a hospital with HCV infection, overall and according to the severity of HCV disease, as well as to assess which of these comorbidities are predictive factors for high-cost patients.

## Methods

### Data source and study population

A population based cohort-study was conducted using data extracted from the hospital discharge database of the Abruzzo and Campania Regions, between January 1^st^, 2007 and December 31^st^, 2015. Each hospital discharge record contains the following data: date of birth, sex, place of residence, date of admission and discharge surgical and other procedures, discharge disposition (i.e. transferred to another facility providing inpatient hospital care, discharged to home, death), one primary diagnosis and up to 5 secondary diagnoses based on the codes of the International Classification of Diseases, Ninth Revision, Clinical Modification (ICD-9-CM). Since this automated system is anonymous, according to Italian Data Protection Authority, neither Ethical Committee approval nor informed consent were required for this study [34]. The anonymous data file is routinely used by the Regional Health Authorities for epidemiological and administrative purposes. The research adhered to the tenets of the Declaration of Helsinki.

We identified all residents in the Abruzzo Region (~1,300,000 inhabitants) and Campania Region (~6,000,000 inhabitants), 18 years and older, who were admitted with a diagnosis HCV code (ICD9-CM 070.41, 070.44, 070.51, 070.54, 070.7, 070.70, 070.71), in primary or secondary diagnoses, between January 1^st^, 2011 and December 31^st^, 2014 (enrollment period). The date of the first hospitalization for HCV at the enrollment was designated as the index date. Patients which also had an ICD9-CM code for HIV-infection (i.e. ICD9-CM 042, V08, 795.71 or 079.53) in primary or secondary diagnoses were excluded. Finally, patients with HCV infection were categorized on the basis of presence or absence of liver complications (LCs) at baseline. LCs were identified using ICD9-CM diagnosis codes recorded in all hospital discharge abstracts collected for each patient (including both ordinary and Day Hospital hospitalizations) during the period of five years preceding the index date (S1 Table). Those included compensated cirrhosis, decompensated cirrhosis, hepatocellular carcinoma and liver transplant. All patients were followed up from the index date up to 1 year (follow-up period).

### Covariates

For each patient, the following variables were assessed at the index date: age group (i.e. 18–54, 55–74 and 75 years and older), sex, HBV co-infection and any comorbidity. The comorbidities were identified using ICD9-CM codes recorded in all hospital discharge abstracts during the period of five years preceding the index date (S1 Table). Cardio- and cerebrovascular disease, diabetes and renal disease were grouped as HCV-related comorbidities since those have been, more recently, demonstrated strongly associated with HCV chronic infection [16, 18–20]. Other comorbidities included into the analysis were: peripheral vascular disease (PVD), paraproteinemias, cancer (excluding HCC), chronic obstructive pulmonary disease (COPD), rheumatic disorders and gastrointestinal (GI) disorders.

### Clinical and economic outcomes

The primary outcome of this study was the incidence of all-cause hospitalizations during the 1-year follow-up. In order to evaluate the incidence of hospitalization for acute event we referred to ordinary hospital admissions only, therefore Day Hospital admissions were excluded.

The secondary outcome was the identification of predictive factors for high-cost patients. To this end, only the sub-sample of patients with at least one hospital admission during the follow-up period was taken into account. High-cost patients were defined as those with total hospitalization costs in the top quintile (i.e., top 20% of costs) based on the distribution of hospital costs during the 1-year follow-up. The remaining 80% of patients were classified as low-cost patients (i.e. bottom 80%).

### Hospitalization resource utilization analyses

Hospitalization resource utilization was assessed by evaluating the following parameters: number of hospital admissions, cumulative length of stay and hospital costs. The number of hospital admissions was calculated as the sum of ordinary admissions within a patient’s 1-year of follow-up period. We defined as cumulative length of stay the total number of days that a patient remained in the hospital during the follow- up period. The hospitalization costs were calculated using the CMS-Disease Related Group (DRG) version 24 grouping algorithm. The DRGs depend on the patients’ ICD9 classification at the time of their discharge from hospital, their age and sex, and the consumption of resources during their hospital stay. According to the DRG-based reimbursement system, every hospitalized patient belongs to a group of diagnostically homogeneous cases. Patients within each category are therefore similar in clinical terms, and expected to require the same level of resources. As a result, patients in the same DRG are assigned the same reimbursement charges. To complete DRG calculation, additional costs due to inpatient stays longer than threshold values used for each DRG were also taken into account. It is important to note that professional fees were not included in this analysis [35]. Hospital costs were adjusted for inflation to 2015 euro.

### Statistical analysis

Baseline characteristics were reported according to disease severity (with and without LCs). Categorical variables were summarized using frequencies and percentages; continuous variables were summarized using means and standard deviations. Negative binomial regression was used to estimate Incidence Rate Ratios (IRRs) for all-cause hospitalizations associated with each clinical and demographic variable, because preliminary exploration of hospitalization count data revealed that the variance was not equal to the mean of the distribution. Incidence rate for all-cause hospitalizations in patients with and without each comorbidity controlling for age group, sex and HBV co-infection, and in patients with HCV-related comorbidities (CMs) equal to 1 and HCV-related CMs ≥ 2, compared to those without HCV-related CMs, were calculated. The results were shown as unadjusted and adjusted IRRs with 95% confidence intervals (CIs). Multivariable logistic regression analysis is used to determine independent predictive factors of high-cost patients. The dependent variable was high-cost patients (top 20%) versus low cost-patients (bottom 80%). Predictors assessed were age, sex, HBV co-infection, liver complications and HCV-related comorbidities. The results were reported as Odds Ratio (OR) with 95% CIs.

All analyses are conducted using STATA software, version 11 (StataCorp., College Station, TX).

## Results

### Characteristics of study cohort

In total, 15,985 patients met the inclusion criteria; of these, 56.0% were male and 44% were between 55 and 74 years old (mean age ±SD was 64.9±15.1 years) (Table 1). At baseline, approximately 19.9% of the HCV cohort had at least one liver complication and nearly half of patients had one or more HCV-related CMs; the most frequent CM was cardiovascular disease, followed by diabetes. In the stratified analysis by disease severity, HCV-patients with LCs were older and had a higher prevalence of HCV-related CMs than those without LCs. In particular, patients with LCs were more likely to have diabetes, COPD, renal disease, GI disorders, paraproteinemias and psychiatric disorders in comparison to those without LCs (Table 1).

**Table 1:** Baseline characteristics of the HCV-cohort overall and stratified by disease severity.

Patient characteristics	Overall (n = 15,985)	Wihout liver complications (n = 12,806)	With liver complications (n = 3,179)	P value
**Male gender (%)**	56.0	54.7	61.6	<0.0001
**Mean age in years** ±**SD**	64.9±15.1	64.1±15.6	67.9±12.1	<0.0001
**Age groups, years (%)**				
18–54	23.9	26.1	15.0	<0.0001
55–74	44.1	42.8	49.0	
≥75	32.0	31.1	35.9	
**HBV co-infection (%)**	2.7	2.2	4.5	
**Liver complications (%)**				
Compensated cirrhosis	9.5	-	47.6	-
Decompensated cirrhosis	8.9	-	44.6	
HCC	5.4	-	27.3	
Liver transplant	1.5	-	7.6	
**Comorbidities (%)**				
Cardiovascular disease	29.4	29.2	29.9	0.44
Diabetes	20.5	18.2	29.7	<0.0001
COPD	19.0	17.9	23.6	0.001
Cerebrovascular disease	16.0	16.1	15.4	0.37
Cancer (excluding HCC)	15.8	14.8	19.8	<0.0001
Renal disease	12.1	11.2	15.8	<0.0001
Peripheral vascular disease	5.4	5.1	6.8	<0.0001
Psychiatric disorders	3.3	3.1	4.3	0.001
Gastrointestinal disease	1.9	1.5	3.1	<0.0001
Other paraproteinemias	1.5	1.1	2.9	<0.0001
Rheumatic disease	0.9	1.0	0.5	0.004
**Number of HCV-related comorbidities (%)**^†^				
0	51.4	53.5	43.0	<0.0001
1	26.9	25.7	31.8	
≥2	21.7	20.8	25.2	

SD,Standard Deviation; HBV, Hepatitis B virus; HCV, Hepatitis C virus; HCC, Hepatocellular carcinoma; COPD, Chronic Obstructive Pulmonary Disease

^†^ HCV-related comorbidities included cardio-and cerebrovascular disease, diabetes and renal disease.

### Clinical outcome

**Fig 1:**
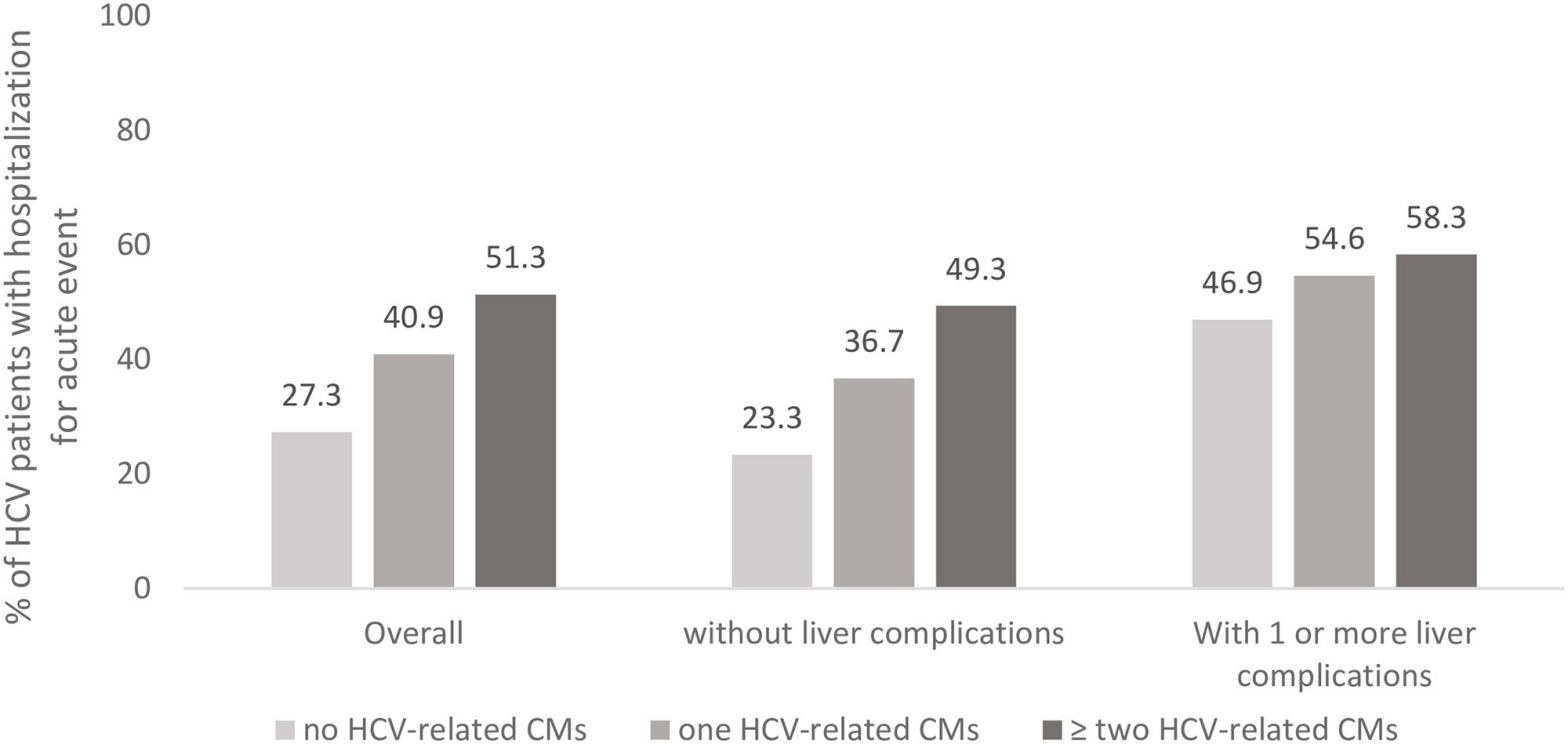
Percentage of HCV patients with hospitalization for acute event during the follow-up stratified by disease severity and HCV-related CMs^†^. CMs, comorbidities. ^†^ HCV-related comorbidities included cardiovascular disease, cerebrovascular disease, renal disease and diabetes.

During the 1-year of follow-up, 36.0% of HCV-infected patients underwent acute hospital admission(s) for any-cause. When compared to individuals without HCV-related CMs, the proportion of admitted patients increased 2.5 times in the presence of two or more HCV-related CMs among HCV patients without LCs and 1.5 times among those with LCs (p<0.0001) (Fig 1).

We examined the risk factors for all-cause hospitalizations in the overall HCV-cohort and separately by those patients with and without liver complication (Table 2). Decompensated cirrhosis and the presence of two or more HCV-related CMs were the strongest predictors of all-cause hospitalization in the overall sample (Table 2). In the sub-cohort of patients without LCs, HBV co-infection and each comorbid condition were associated with a significantly increased risk of hospitalization(s) compared to those without co-infection or each comorbidity class; whereas among patients with LCs, only HBV co-infection, cardiovascular disease, diabetes, COPD, cancer and LCs themselves were the predictors of all-cause hospitalizations (Table 2).

**Table 2:** Incidence Rate Ratio (IRR) of all-cause hospitalizations.

	Overall Adjusted IRR (CI 95%)	Without liver complications Adjusted IRR (CI 95%)	With liver complications Adjusted IRR (CI 95%)
**Male gender (Ref Female)**	1.06 (1.00–1.12)	1.04 (0.97–1.11)	1.07 (0.96–1.19)
**Age, years (ref 18–54)**			
55–74	1.00 (0.93–1.08)	1.07 (0.98–1.17)	0.74 (0.64–0.86)
≥75	1.01 (0.93–1.10)	1.11 (1.01–1.23)	0.66 (0.56–0.78)
**HBV co-infection (ref no HBV co-infection)**	**1.28 (1.09–1.49)**	**1.29 (1.05–1.59)**	**1.19 (1.03–1.37)**
**Liver complications (ref no liver complication)**			
Compensated cirrhosis	**1.39 (1.28–1.52)**	-	**1.19 (1.03–1.37)**
Decompensated cirrhosis	**1.65 (1.47–1.85)**	-	**1.49 (1.32–1.68)**
HCC	**1.36 (1.21–1.53)**	-	**1.31 (1.17–1.50)**
Liver transplant	**0.64 (0.50–0.80)**	-	**0.56 (0.45–0.70)**
**Comorbidities**^†^ **(ref no comorbidity)**			
Cardiovascular disease	**1.42 (1.33–1.51)**	**1.47 (1.36–1.58)**	**1.25 (1.12–1.40)**
Diabetes	**1.23 (1.15–1.32)**	**1.27 (1.17–1.38)**	**1.14 (1.02–1.27)**
Chronic Obstructive Pulmonary Disease	**1.31 (1.23–1.40)**	**1.37 (1.26–1.48)**	**1.17 (1.05–1.32)**
Cerebrovascular disease	**1.20 (1.11–1.28)**	**1.21 (1.11–1.32)**	1.12 (0.97–1.28)
Cancer (excluding HCC)	**1.73 (1.62–1.86)**	**1.90 (1.76–2.10)**	**1.38 (1.23–1.54)**
Renal disease	**1.41 (1.31–1.53)**	**1.53 (1.39–1.67)**	1.14 (1.00–1.31)
Peripheral vascular disease	**1.18 (1.06–1.32)**	**1.22 (1.06–1.39)**	1.15 (0.95–1.40)
Psychiatric disorders	**1.31 (1.23–1.63)**	**1.51 (1.27–1.80)**	1.17 (0.93–1.48)
**Number of HCV-related Comorbidities**^‡^**(ref 0)**			
1	**1.45 (1.36–1.55)**	**1.39 (1.30–1.48)**	1.04 (0.96–1.13)
≥2	**1.96 (1.82–2.11)**	**1.79 (1.67–1.93)**	**1.16 (1.06–1.27)**

CI, Confidence interval; HBV, Hepatitis B virus; HCV, Hepatitis C virus; HCC, Hepatocellular carcinoma.

^†^ Comorbidities accounting for less than 3% were not included in the model.

^‡^ HCV-related comorbidities included cardio-and cerebrovascular disease, diabetes and renal disease.

Bold values indicate statistical significance.

#### Hospitalization resource utilization and predictive factors for high cost-patients

**Fig 2:**
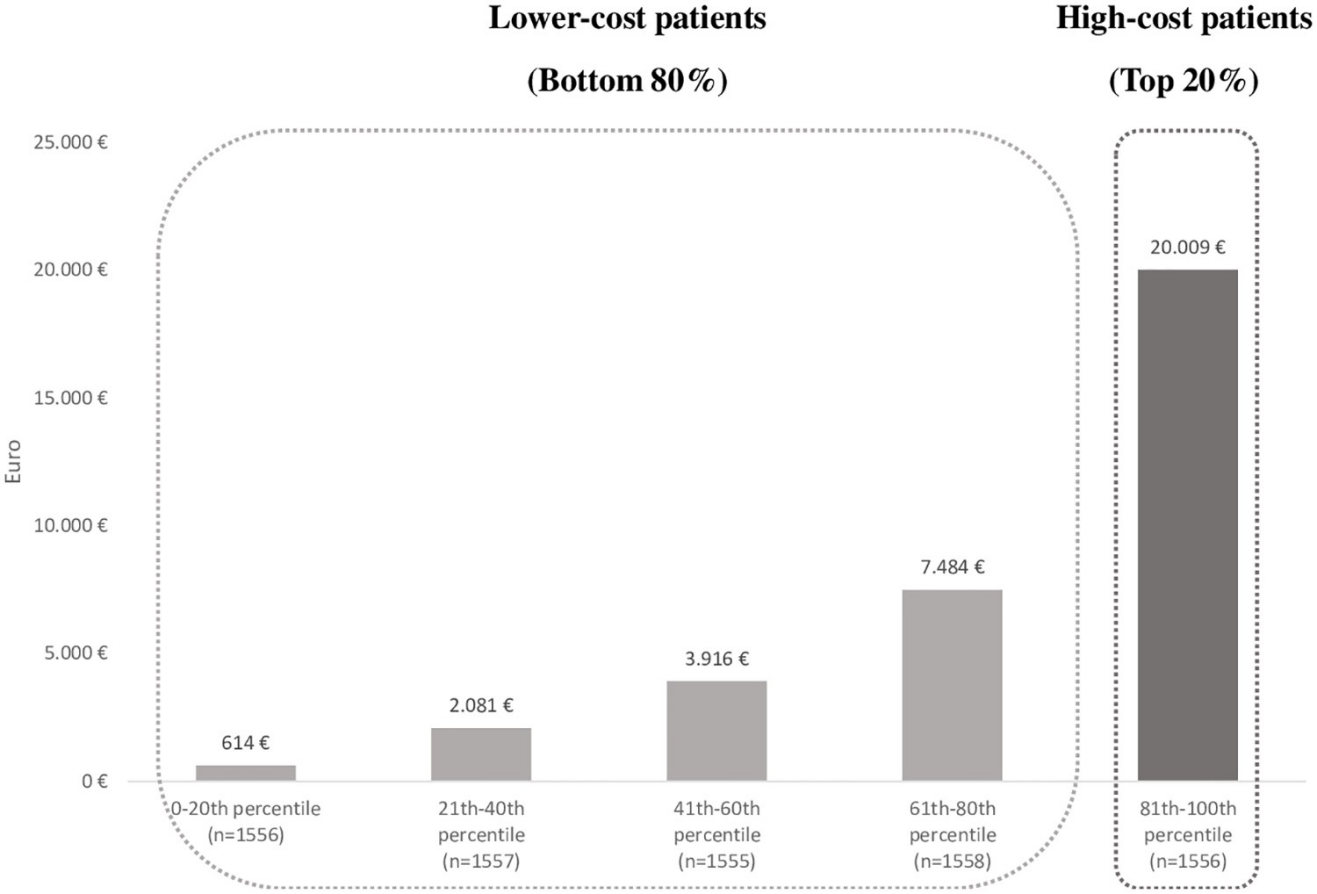
Mean per patient hospitalization costs during the follow-up period.

The sub-sample of patients with at least one hospital admission during the follow-up (Day Hospital or acute hospital admission) included 7,782 individuals, which accounted for a total annual spending of EUR 53 million. Of these, 1,556 were high cost-patients (top 20%), and the remaining 6,226 low cost-patients (bottom 80%) (S1 Fig). Baseline characteristics of HCV cohort stratified by hospitalization resource utilization were reported in the S2 Table. High-cost patients accounted for approximately 58% of the total hospitalization costs (over EUR 31 million). The mean cost per patient in the top quintile (mean cost EUR 20,009) was approximately 2.5 times higher than that of patients in the first four cost quintiles (mean cost EUR 7,484) and nearly 32 times higher than the first cost quintile (mean cost EUR 614) (Fig 2). As shown in Table 3, the number of acute admission(s) per patient, the mean cumulative length of stay and the proportion of patients with in-hospital death were all significantly higher in high-cost patients compared to the low-cost patients.

**Table 3:** Hospital utilization, outcome and inpatient costs in Euros during 1-year of follow-up.

	Lower-cost patients (N = 6,226)	High-cost patients (N = 1,556)
Number of hospitalization for acute event, mean ± SD	1.0±0.9	3.2±2.4
Cumulative length of stay, mean ± SD	7.4±10.0	41.0±41.5
Inhospital death, %	5.2	13.7
Total hospital cost per patient-year mean ± SD	3,524±2,706	20,009±12,941

SD, Standard Deviation.

Multivariate logistic regressions showed that LCs (excluding liver transplant) were the strongest predictor of high hospitalization costs (Table 4). The ORs of being in the top 20% of hospitalization costs for HCV patients were 1.30 (95% CI:1.08–1.55), 1.47 (95% CI:1.22–1.77) and 1.82 (95% CI:1.48–2.23) in the presence of compensated cirrhosis, DC and HCC, respectively; while those of patients diagnosed with 1 or ≥ 2 HCV-related CMs were 1.37 (1.19–1.58) and 1.63 (1.40–1.90), respectively. Similarly, the presence of cancer and cardiovascular disease increased the likelihood of being in the top 20% of hospitalization costs by 49% and 37%, respectively. Other factors independently associated with highest hospitalization costs were cerebrovascular disease, PVD and renal disease (Table 4).

**Table 4:** Multiple logistic regression model to identify predictors of high-cost HCV patients.

	Odds Ratio^†^	95% CI
**Male gender (Ref Female)**	1.12	1.00–1.26
**Age, years (ref 18–54)**		
55–74	1.02	0.87–1.21
≥75	0.98	0.81–1.17
**HBV co-infection (ref no HBV co-infection)**	1.06	0.78–1.45
**Liver complications (ref no liver complication)**		
Compensated cirrhosis	**1.30**	**1.08–1.55**
Decompensated cirrhosis	**1.47**	**1.22–1.77**
HCC	**1.82**	**1.48–2.23**
Liver transplant	**0.55**	**0.36–0.86**
**Comorbidities**^‡^ **(ref no comorbidity)**		
Cardiovascular disease	**1.37**	**1.20–1.57**
Diabetes	1.10	0.96–1.26
Chronic Obstructive Pulmonary Disease	1.04	0.90–1.19
Cerebrovascular disease	**1.17**	**1.01–1.37**
Cancer	**1.49**	**1.30–1.71**
Renal disease	**1.25**	**1.07–1.45**
Gastrointestinal disease	1.06	0.83–1.35
Peripheral vascular disease	**1.26**	**1.02–1.56**
Psychiatric disorders	1.04	0.90–1.19
**Number of HCV-related comorbidities (ref 0)**^§^		
1	**1.37**	**1.19–1.58**
≥2	**1.63**	**1.40–1.90**

CI, Confidence interval; HBV, Hepatitis B virus; HCV, Hepatitis C virus; HCC, Hepatocellular carcinoma.

^†^ Odds Ratio for high cost patients (top 20%) compared with lower-cost patients (bottom 80%) (i.e., high-cost = 1, lower-cost = 0 was the dependent variable). Bold values indicate statistical significance.

^‡^ Comorbidities accounting for less than 3% were not included in the model.

^§^ HCV-related comorbidities included cardio-and cerebrovascular disease, diabetes and renal disease.

## Discussion

The landscape of medical needs and related healthcare costs due to HCV infection underwent deep changes in recent decades; in parallel, a sharp increase in the knowledge of the factors causing HCV-related disease progression was acquired [12, 18, 19, 33]. Most estimates indicate that the burden of HCV disease is due to increase world-wide in the next 2 decades, in the absence of comprehensive State-run interventions to treat known HCV infections. Screening at- risk populations for HCV and, immediate DAA treatment for most of hidden HCV disease before the overt clinical emergence of HCV-related complications are needed [29–32]. The most current guidelines recommend treatment for all HCV-patients, and when prioritization is needed, they identify patients with extrahepatic manifestations as a priority to minimize future morbidity and mortality [36, 37]. In addition to estimates related to HCV disease burden, an extensive body of evidence has been recently generated. In fact, these new evidences demonstrate the significant clinical and economic consequences of extrahepatic HCV manifestations [13, 14]. In particular, due to the pleiotropic effects of HCV replication, including endothelial activation and consequently vascular instability, the role of HCV in increasing cardio- and cerebrovascular morbidity and mortality has been recently established by several pivotal epidemiological investigations [16, 18, 38]. Similarly, more evidence has been gathered in recent years to indicate that diabetes mellitus may influence and accelerate the rate of hepatic complications in HCV-infected patients [20, 39].

As a consequence, the assessment of the inpatient burden related to HCV disease may be valuable in order to define strategies related to HCV screening and treatment in each State; in view of the current availability of DAA treatments these strategies may in principle enable stakeholders to aim for HCV elimination [29–31]. This scenario is particularly relevant in territories with high HCV endemicity as demonstrated in the both Abruzzo and the Campania Italian regions [38, 40–43]. On the basis of these considerations and in the absence of any recent estimate about medical expenses caused by hepatic and extra-hepatic complications of HCV disease in these Italian regions, we planned the current study, aimed to describe the characteristics of the cohort of HCV-infected patients revealed by hospital-based available records, and quantified the effect of these characteristics on hospital-related outcomes and costs.

First of all, we studied a total of 15,985 HCV-infected patients which account for a hospital spending of EUR 53 million during 1-year of follow-up, although involving only 0.2% of resident population. In accordance with many other reports, the highest HCV prevalence was found in patients over 50 years of age [1, 44]. It is well known that chronic HCV infection causes more complications when long lasting and cooperating with aging in influencing the progression of liver fibrosis and the development of HCC [7, 8, 12, 45, 46]. In line with the figures depicted by other studies, our data revealed that around 20% of patients had a severe liver-related complication (9% DC and 5% HCC) at baseline [5, 6]. Interestingly, in spite of one third of patients in our sample being over 75 years, the age of most of the remaining two thirds was between 55 and 74 years, which is a very good target for preventative measures of intervention. Among these, the 80% of HCV-infected subjects were at risk of developing advanced liver disease, and represented even more an ideal setting for preventative interventions, first of all through early treatment with antiviral therapy [8, 11, 23, 45]. This evidence was strengthened by our approach of assessing characteristics associated with patients who are likely to utilize more healthcare resources. Indeed, as expected our data revealed that the distribution of total hospitalization costs was markedly skewed among HCV patients. In accordance with previous studies, we found that high-cost HCV patients were more likely to have severe liver complications and HCV-related CMs [47, 48]. HCC is the strongest predictor of becoming a high-cost healthcare user, suggesting that as disease severity increases, the more costly and resource consuming do patients become [1, 2, 7, 10, 20, 22, 31, 49]. These findings highlight the potential for the remarkable benefits of deploying mass screening of HCV infected, unaware patients, mostly aged 55 to 74 years of age, among at risk populations. Antiviral treatment for such patients would have the chance of rapidly reducing the quantity of HCV patients consuming most hospitalization resources in the current heath care scenario of both Regions.

Moreover, in accordance with other studies we provide additional evidence that hospitalization risk and costs were driven by extrahepatic complications in addition to liver-related sequelae [13, 19, 38, 50]. Indeed, we found that the presence of two or more HCV-related CMs (cardiovascular disease, diabetes, cerebrovascular disease and renal impairment) increased 2-fold the risk of occurring in hospital admission and rose by 63% the likelihood of becoming high-cost patients when compared with those without HCV-related CMs. In particular, cardiovascular disease increased by 42% the risk of hospitalization in the overall sample (25% and 47% in HCV patients with and without LCs, respectively). In line with other recent lines of evidence, this would suggest that HCV patients with CV disease may be prioritized over other as they are likely to incur more cost [16, 18, 38]. Identifying and early treatment of HCV patients in this large population setting would be an innovative way of addressing local HCV screening policies and may lead to substantial savings in healthcare costs related to extrahepatic conditions in our Regions [2, 13, 14, 38].

The current study is subject to several limitations. First of all, data were retrieved from a large administrative database, providing no laboratory and instrumental information on the enrolled patients. As a consequence, clinical conditions of hepatic and extrahepatic disease could be only deduced by available diagnostic codes which might have caused an underestimate. In addition, given the study design, it is not possible to establish whether or not HCV preceded the development of these comorbidities. Second, our cost-analysis were based only on DRG tariffs that have nonetheless been frequently associated with a relevant underestimation of real costs of patients [35]. Indeed, no estimates of personnel costs and drug expenses could be neither derived by our search, nor guessed indirectly. Finally, generalizations of the study’s findings should be made with caution, particularly because data are limited to the hospital setting of two Italian regions. Since we analyzed a cohort of admitted HCV patients, the risk of hospitalization in our cohort is much more likely than in HCV patients who do not require hospitalization in the first place. In spite of that, the strength of our study is the use of a large-scale population-based cohort in territories with high HCV endemicity and the ability to draw information from a real-world setting through the analysis of administrative data.

In conclusion, we provide an additional line of evidence that chronic HCV infection is associated with a relevant inpatient burden in two Italian regions with high prevalence of HCV infection. Since the majority of patients were without liver complications at baseline, liver disease progression alone did not fully predict high resource utilization. Beyond advanced liver disease, HCV-related comorbidities (especially cardiovascular disease) are strong predictors of high hospitalization costs. In particular, in non-advanced HCV disease, extrahepatic HCV comorbidities significantly increased the risk of hospitalization, mostly in an additive manner. This highlights the potential benefit that the early identification and treatment of HCV infected patients might have on the reduction of hospitalization costs driven by HCV related extrahepatic complications. We therefore provide an additional line of evidence that identifying unaware HCV-patients with extrahepatic manifestations may reduce HCV related morbidity and mortality beyond the benefits of reducing HCV related liver morbidity and mortality, thus reducing heath care costs in parallel. In accordance with the most current guidelines, it is important to identify HCV-patients with extrahepatic manifestations as a priority to minimize future morbidity and mortality [36]. Healthcare stakeholders may therefore benefit from new, local and detailed evidence when planning future interventions to track and reduce HCV related disease burden in our Regions and around the world.

## Supporting information

S1 Table Codes used to identify comorbid conditions. (DOCX)

S2 Table Baseline demographic and clinical characteristics of HCV cohort stratified by hospitalization resource utilization during 1-year of follow-up. (DOCX)

S1 Fig Identification of high-cost patients. (TIF)

S1 Dataset (ZIP)
