# Supplementary material for: The inpatient hospital burden of comorbidities in HCV-infected patients: A population-based study in two Italian regions with high HCV endemicity (The BaCH study)
Source: PLoS One. 2019 Jul 10;14(7):e0219396. doi: 10.1371/journal.pone.0219396 (PMC6619769; doi:10.1371/journal.pone.0219396)
Supplement: S1 Table — (DOCX) [file pone.0219396.s001.docx]

**S1 Table. Codes used to identify comorbid conditions.**

| Clinical condition | ICD9 Diagnostic codes | Procedure ICD9 codes |
| --- | --- | --- |
| HBV | 070.2x, 070.3x |  |
| Diabetes | 250xx, 357.2, 362.0x, 366.41 |  |
| Cardiovascular disease | 410.xx, 411.xx ,413.xx, 414.xx ,427.5,412,  426.xx, 427.0, 427.1, 427.2, 427.3x, 427.4, 427.6x, 427.8x, 427.9  428.x, 785.5x, 402.01, 402.11, 402.91, 404.01, 404.03, 404.11, 404.13, 404.91, 404.93 ,429.2, 440.xx, V45.81, V45.82 | 36.0x, 36.1x,36.2, 36.3, 36.9, 88.5x,37.8x, 38.12 |
| Cerebrovascular disease | 430xx-438xx | 38.11, 38.12 |
| Peripheral vascular disease | 250.7x, 441.xx, 442.xx, 443.xx, 444.xx, 447.1 | 38.13, 38.14, 38.16, 38.18, 39.25, 39.29, 39.50, 39.90, 99.10 |
| Renal disease | 016.0; 095.4; 189.0; 189.9; 223.0; 236.91; 250.4; 271.4; 274.1; 283.11; 403.X1; 404.X2; 404.X3; 440.1; 442.1; 447.3; 572.4; 580-588xx; 591; 642.1; 646.2; 753.12-753.17; 753.19; 753.2; 794.4; V45.11 |  |
| Cirrhosis | 571.2, 571.5, 571.6 |  |
| Decompensated cirrhosis | 456.0, 456.20, 456.1, 456.21, 572.2, 572.3, 572.4, 789.5, 789.59 |  |
| Liver transplant | 50.5, 50.51, 50.59, V42.7 |  |
| Hepatocellular carcinoma | 155.0 |  |
| Other paraproteinemias | 273.2 |  |
| Gastrointestinal disease | 530.7; 531-534; 569.84; 569.85; 578 |  |
| Cancer (excluding HCC) | 140xx-208xx; 230xx-231xx; 233xx-234xxxx  (excluding 155.0) |  |
| Rheumatic disease | 714.xx |  |
| Psychiatric conditions | 296.20-296.25, 296.30-296.36, 296.5, 206.6, 296.82, 311xx, 296.90, 309.0, 309.1, 309.28, 309.81, 308.3, 309.24, 300.00-300.02 |  |
| Chronic Obstructive Pulmonary Disease | 491-494; 496; 510 |  |

HBV, Hepatitis B virus; HCC, Hepatocellular carcinoma.
