# Supplementary material for: The inpatient hospital burden of comorbidities in HCV-infected patients: A population-based study in two Italian regions with high HCV endemicity (The BaCH study)
Source: PLoS One. 2019 Jul 10;14(7):e0219396. doi: 10.1371/journal.pone.0219396 (PMC6619769; doi:10.1371/journal.pone.0219396)
Supplement: S2 Table — (DOCX) [file pone.0219396.s002.docx]

**S2 Table. Baseline demographic and clinical characteristics of HCV cohort stratified by hospitalization resource utilization during 1-year of follow-up.**

|  | Patients without hospitalization costs  (N=8203) | Lower-cost  patients  (N=6226) | High-cost  patients  (N=1556) |
| --- | --- | --- | --- |
| **Male gender (%)** | 54.4 | 56.9 | 61.6 |
| **Age groups (%)** |  |  |  |
| 18-54 | 26.8 | 21.8 | 17.2 |
| 55-74 | 42.4 | 45.7 | 46.7 |
| ≥75 | 30.9 | 32.5 | 36.7 |
| **HBV co-infection (%)** | 2.1 | 3.2 | 3.6 |
| **Liver complications (%)** | 13.8 | 23.7 | 36.8 |
| Compensated cirrhosis | 7.2 | 11.1 | 15.3 |
| Decompensated cirrhosis | 5.6 | 10.6 | 18.8 |
| HCC | 3.2 | 6.2 | 14.0 |
| Liver transplant | 0.9 | 2.3 | 1.6 |
| **Comorbidities (%)** |  |  |  |
| Cardiovascular disease | 25.4 | 31.8 | 40.6 |
| Diabetes | 16.9 | 23.1 | 29.0 |
| Chronic Obstructive Pulmonary Disease; | 15.8 | 21.6 | 26.0 |
| Cerebrovascular disease | 14.4 | 16.8 | 20.8 |
| Cancer | 10.6 | 19.6 | 27.7 |
| Renal disease | 9.0 | 14.6 | 18.8 |
| Peripheral vascular disease | 3.9 | 6.3 | 9.7 |
| Psychiatric disorders | 2.6 | 3.8 | 3.9 |
| Gastrointestinal disease | 2.2 | 1.9 | 3.3 |
| Other paraproteinemias | 1.1 | 1.7 | 2.4 |
| Rheumatic disease | 0.9 | 0.8 | 1.4 |
| **Number of HCV-related comorbidities**^†^ **(%)** |  |  |  |
| 0 | 57.3 | 53.0 | 36.4 |
| 1 | 25.3 | 26.4 | 31.3 |
| ≥2 | 17.4 | 20.6 | 32.3 |

HBV, Hepatitis B virus; HCV Hepatitis C virus; HCC, Hepatocellular carcinoma.

^†^ HCV-related comorbidities included cardio-and cerebrovascular disease, diabetes and renal disease.
