# Supplementary figures and images for: The inpatient hospital burden of comorbidities in HCV-infected patients: A population-based study in two Italian regions with high HCV endemicity (The BaCH study)
Source: PLoS One. 2019 Jul 10;14(7):e0219396. doi: 10.1371/journal.pone.0219396 (PMC6619769; doi:10.1371/journal.pone.0219396)

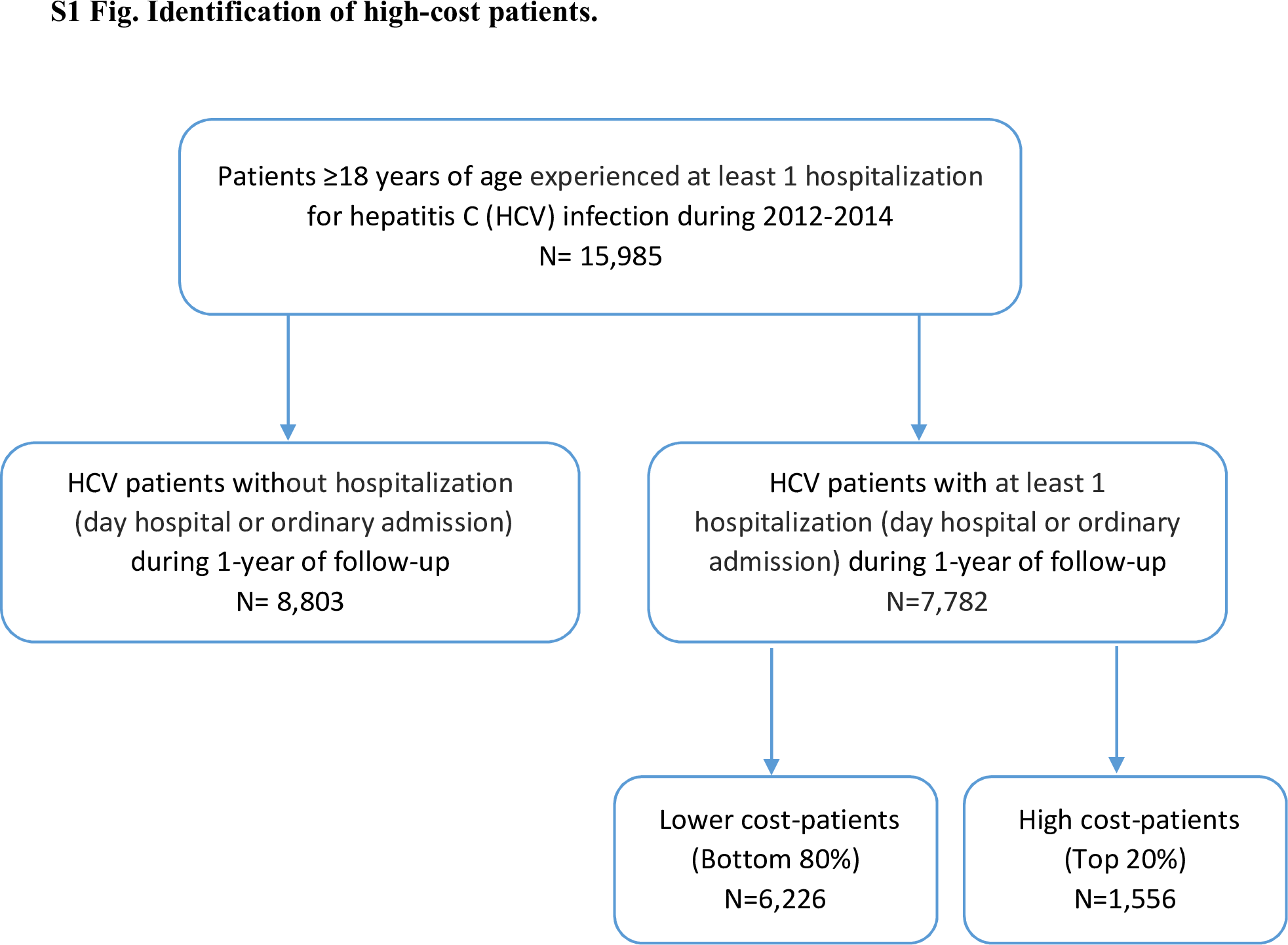

Supplement: S1 Fig — (TIF) [file pone.0219396.s003.tif]
